# Radon Concentration Survey in Settlements Located in Uranium Mining Territory in Northern Kazakhstan

**DOI:** 10.3390/ijerph22050723

**Published:** 2025-05-02

**Authors:** Yerlan Kashkinbayev, Danara Ibrayeva, Moldir Aumalikova, Elena Saifulina, Dinara Bizhanova, Elvira Mussayeva, Aigerim Shokabayeva, Madina Kairullova, Anel Lesbek, Baglan Kazhiyakhmetova, Meirat Bakhtin

**Affiliations:** International Research Institute of Radiobiology and Radiation Protection, nJSC Astana Medical University, Astana 010000, Kazakhstan; kashkinbaev.ye@gmail.com (Y.K.); aumalikova.m@amu.kz (M.A.); saifulina.e@amu.kz (E.S.); bizhanova.d@amu.kz (D.B.); mussayeva.e@amu.kz (E.M.); shokabaeva.a@amu.kz (A.S.); kairullova.m@amu.kz (M.K.); omar.a@amu.kz (A.L.); kazhiyakhmetova.r@amu.kz (B.K.); bakhtin.m@amu.kz (M.B.)

**Keywords:** uranium mining, radon exposure, equivalent equilibrium volume activity, radiation exposure risks

## Abstract

Among the Central Asian countries, Kazakhstan is experiencing significant growth in uranium production and plays a key role in the mining industry. The aim of this study was to assess environmental gamma radiation levels and indoor radon concentrations in the settlements of Aqsu, Saumalkol, and Arykbalyk—situated in regions with a history of uranium mining activities—to evaluate potential radiation exposure risks to the local population. Measurements of ambient gamma radiation dose rates indicated that Saumalkol exhibited the highest variability, with recorded values reaching up to 0.56 ± 0.19 µSv/h, suggesting potential influence from abandoned mining areas. The equivalent equilibrium volume activity of radon revealed severe contamination in Aqsu (mean: 303 ± 57 Bq/m^3^, max: 4974 Bq/m^3^) and Saumalkol (mean: 658 ± 114 Bq/m^3^, max: 2470 Bq/m^3^). These findings underscore the need for immediate intervention measures such as improved ventilation and radon mitigation strategies to reduce exposure risks and protect residents from radiation-induced health hazards. This study presents a screening method to identify areas with potential radon risks. However, radon dose assessment requires long-term measurements for accurate evaluation of exposure levels and health risks, with extended monitoring needed for comprehensive assessment.

## 1. Introduction

Environmental radiation originates from many naturally occurring and man-made sources. Radioactive nuclides such as uranium, thorium, and potassium occur naturally everywhere in the environment. The largest proportion of human radiation exposure comes from natural sources [1,2].

More than half of the population’s radiation dose of natural origin comes from radon [3]. Since most of our time is spent indoors, measuring and limiting radon concentration in buildings is important [4,5]. The primary sources of radon in premises, in order of importance, are: the soil beneath the building, the building materials, the outside air, and water from private wells. The soil under the building is by far the most significant source of indoor radon [6]. Radon (^222^Rn) is one of the intermediate products of ^238^U. Uranium is found to some extent in all rocks and soils. ^222^Rn is the gaseous radioactive product of the decay of ^226^Ra. Some atoms of this radon isotope are released from the solid matrix of the material by recoil when ^226^Ra decays. Radon atoms entering the pore space are then transported in various ways until they either decay or are released into the atmosphere (exhalation) [7,8,9].

Among the Central Asian countries, Kazakhstan is experiencing significant growth in uranium production and plays a key role in the mining industry. It is estimated that approximately 20% of the world’s uranium reserves are in Kazakhstan. Active uranium mining is the responsibility of the National JSC KAZATOMPROM. According to a compilation made in 1993, there were 127 legacy sites left behind by uranium mining/milling of the past. Geographically, the legacy sites are located in three areas: Northern Kazakhstan: 12 deposits—sites in the Kokshetau area, the Nos 8, 9, 1, 3, and 12 group of mines (Kosatchinnoe, Shatskoe, Glubinnoe, Agashskoe, and Koksorskoe), and Manybaiskoe and the Stepnogorsk Mining Chemical Processing plant site with tailings (the total amount of waste = 81.2 Mt); Southern/Central Kazakhstan: 4 deposits—sites Kurdai, Vostochniy, and Zapadniy in the Zhambyl region (Southern Kazakhstan), and Karasaiskiy and Ulken-Akzhal in Central Kazakhstan (the total amount of waste = 117.8 Mt); and Western Kazakhstan: 2 deposits—the Koshkar Ata site with tailings (near Aktau city) (the total amount of waste = 58.9 Mt) [10,11,12].

In recent years, Kazakhstan has conducted comprehensive evaluations of radioactivity at its uranium mines and processing facilities. These assessments included extensive surveys to measure gamma dose rates and indoor radon concentrations. The results indicate that the effective radiation dose for individuals living near uranium mine tailings and nuclear facilities can exceed 20–30 mSv/y, surpassing the recommended public exposure limit set by the International Commission on Radiological Protection (ICRP). Such elevated exposure levels are associated with increased risks of lung and stomach cancers, as well as other radiation-induced health effects, particularly due to prolonged inhalation of radon and ingestion of contaminated water. In addition to human health impacts, the accumulation of radionuclides in soil and water can disrupt local ecosystems, contaminate food chains, and affect agricultural productivity. Surveys of uranium mining areas in the Stepnogorsk region of Northern Kazakhstan revealed notable radionuclide enrichment, with radioactive anomalies in residential zones exceeding global averages [13,14,15,16].

Similar studies conducted in other uranium mining regions around the world have reported comparable environmental and public health concerns. For example, in the Czech Republic and Romania, legacy uranium mines have been linked to elevated indoor radon concentrations and long-term groundwater contamination, posing chronic exposure risks to local residents [17,18]. In the United States, particularly in the Navajo Nation, historical uranium mining has resulted in widespread soil and water contamination, leading to increased cancer rates and prompting large-scale environmental health studies and remediation programs [19]. Likewise, in Namibia and South Africa, uranium-rich geological formations combined with poor mine waste management have led to radon exhalation and radionuclide migration into agricultural zones and residential areas [20,21]. These international examples demonstrate that legacy uranium mining poses persistent risks that require targeted monitoring and risk assessment—making similar investigations in Kazakhstan both timely and essential.

The aim of this study was to assess environmental gamma radiation levels and indoor radon concentrations in the settlements of Aqsu, Saumalkol, and Arykbalyk—situated in regions with a history of uranium mining activities—to evaluate potential radiation exposure risks to the local population.

The equivalent equilibrium volume activity (EEVA) is the weighted sum of the volume activities of the short-lived progeny of radon isotopes—^218^Po, ^214^Pb, ^214^Bi; ^212^Pb, and ^212^Bi. The EEVA is a derived measure used to estimate the potential alpha energy concentration of radon and its progeny in air, assuming radioactive equilibrium between radon and its short-lived decay products. It provides a standardized way to assess exposure levels for health risk evaluation, particularly in indoor environments where equilibrium conditions may not naturally occur. By comparing the measured values with established intervention levels and safety limits set by international regulatory bodies such as the ICRP [7,22,23,24,25], this study aimed to identify high-risk areas. The findings will support recommendations for necessary mitigation measures to reduce radiation exposure and protect public health.

## 2. Materials and Methods

### 2.1. Study Area

**Figure 1:**
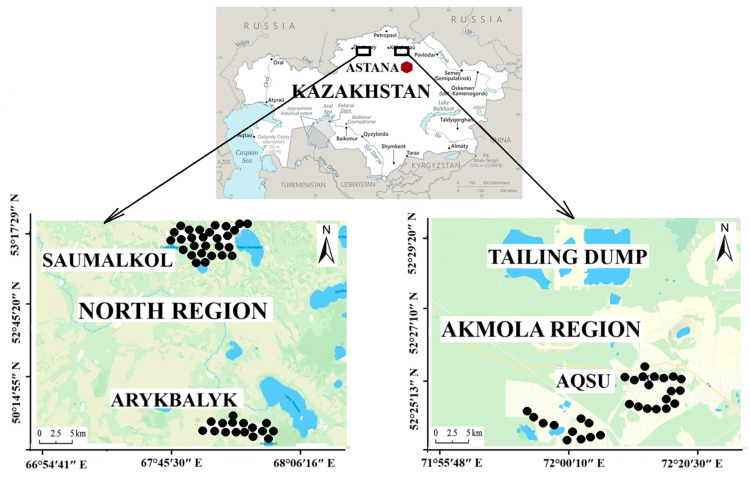
Map of settlement locations.

The survey was carried out in three settlements near uranium mining projects in different regions of Northern Kazakhstan. The settlements were chosen so that they covered the different types of uranium mining projects in Northern Kazakhstan with different sources of contamination. Northern Kazakhstan consists of two main regions: Akmola and Northern Kazakhstan (Figure 1). The Akmola region is known for its significant uranium mining industry and includes the Aqsu settlement. The region has experienced environmental impacts due to prolonged mining operations and is one of the sites with high radiation risk. Aqsu has been influenced by the mining sector viz., uranium and gold mining, which plays a crucial role in the local economy. Because the mine and milling plant are close to living areas and the settlement border, the radioactive and chemical pollution includes ground and surface water, agricultural land, roads, and living areas. The tailing dump of the Stepnogorsk Mining and Chemical Plant (SMChP) was established in 1956, and built in stages. Some uranium-related activity is ongoing today, as the plant still extracts uranium from ores that are present throughout the country at old sites with water outflow, and these are delivered to the SMChP for processing into yellow cake. The settlement is conditionally separated in two districts and one of the largest districts is Kvartsitka. In these regions the houses are old and were built with basements (before 1950).

The Akmola region has a continental climate, with cold winters and hot summers, making it suitable for agriculture despite being an industrial zone. The uranium mining sector is a key economic driver, but it also raises concerns regarding environmental safety and radiation levels [26].

In the north of the country, up until the mid-1990s, uranium deposits were developed by five mining administrations of the former Tselinny Mining and Chemical Combine (now SMChC). Uranium Mine No. 12 in the Northern Kazakhstan region had been explored back in the 1970s. The industry was managed from Moscow, so the collapse of the Soviet Union and the drop in global uranium prices led to uranium extraction becoming unprofitable and eventually ceasing altogether, with the mines left to decay on their own.

However, the issue of abandoned and radiation-hazardous uranium mines did eventually attract the attention of the leadership of the Republic of Kazakhstan (RK). In December 1999, by decision of the Government of the RK, the state enterprise “Uranlikvidrudnik” was established. Along with this, a program was launched for the conservation of uranium mining enterprises and the elimination of the consequences of uranium deposit development for the years 2001–2010. During this period, two uranium deposits in the north of the country were liquidated, and three were conserved. The shafts were filled with concrete, the territory was fenced off, and signs were installed warning that approaching the area was hazardous to health.

Having completed its mission, in 2010, “Uranlikvidrudnik”, itself, was liquidated. After it ceased operations, the sites were left without any oversight. The fences were scrapped for metal by local villagers, and buildings began to be dismantled for construction materials. Radiation-contaminated materials are now being used in everyday construction and local industries [27].

The Northern Kazakhstan region includes the Saumalkol and Arykbalyk settlements, which are located near an abandoned mine and a former uranium mining area, respectively. Saumalkol’s location near a historically significant uranium mining area presents economic opportunities and environmental challenges. The region is primarily agricultural and is known for its wheat production and livestock farming. However, abandoned industrial sites pose challenges for sustainable land use. Both areas are strategically important due to their natural resources, agricultural potential, and historical mining activities. The legacy of uranium extraction has implications for the settlement’s health, environment, and socio-economic conditions [27].

### 2.2. Measurement of Ambient Equivalent Dose Rates

The ambient gamma equivalent dose rates, H* (10), were measured at all designated sampling locations within the settlement using the RKS-01-SOLO radiometer–dosimeter, manufactured by SOLO LLP, Republic of Kazakhstan. This portable device is specifically designed to detect and quantify ionizing radiation, particularly gamma radiation. The RKS-01-SOLO dosimeter operates with fixed time intervals (1–60 s) and is simultaneously associated with the locality information (latitude and longitude; precision: 10 m), allowing it to detect ambient gamma dose rates within a broad range of 0.01 to 15 × 10^6^ µSv/h, with a scintillation detector.

Approximately 15 readings were collected at various points within each settlement (Table 1). At each measurement point, five repeated measurements were carried out to reduce statistical fluctuations and improve data reliability. The arithmetic mean of these five readings was calculated and recorded as the representative dose rate value for that location. The standard deviation of the five measurements was determined to assess the spread of the data, and the error of the mean was calculated following standard statistical methods for uncertainties. The typical measurement uncertainty of the instrument for gamma radiation is ±12% [28].

The measurements were conducted during the summer (July–August) and autumn (September–November) seasons of 2024. This seasonal coverage was intended to capture potential variations in radon concentrations due to environmental and climatic factors.

All measurements were conducted following the International Atomic Energy Agency (IAEA) guidelines, ensuring compliance with internationally recognized radiation monitoring standards [29]. The gamma radiation levels were systematically recorded both indoors and outdoors at a standardized height of 1 m above ground level.

### 2.3. Radon Concentration Measurement

The equivalent equilibrium volume activity (EEVA) of radon in indoor air was measured using the automatic compact radiometer, Alpharad Plus, manufactured by DOZA SPZ, Russian Federation. The Alpharad Plus operates using a semiconductor silicon detector, which is designed to accurately measure the alpha particle energy emitted by the radioactive decay of radon and its thoron progeny. This allows for precise quantification of radon concentrations in the air. The Alpharad Plus has a wide measurement range for radon concentration (Radon-222), spanning from 1 to 2 × 10^6^ counts per second (cps). This range ensures that both low-level background concentrations and elevated radon levels in high-risk areas can be effectively detected. The Alpharad Plus has a relative uncertainty of ±30% for radon EEVA measurements. This uncertainty accounts for counting statistics, calibration accuracy, environmental factors, and instrumental sensitivity.

At each location, the measurement process took approximately 2 h. This included a 30 min radon accumulation period, followed by two consecutive 20 min measurement cycles. Air was pumped into the measurement chamber before each cycle to ensure sample quality. Measurements were performed indoors in living spaces such as bedrooms or living rooms, where occupants typically spend extended periods. The device was placed at least 50 cm away from walls, at least 150 cm above the floor (breathing-zone height), and at least 1 m away from windows, ventilation outlets, or heaters. All measurements were conducted in strict accordance with [30], which outlines best practices for radon detection in ambient air. Adherence to these guidelines ensured consistency, accuracy, and international comparability of results.

Sampling locations were selected based on their proximity to former uranium and gold mining sites, with a focus on buildings near tailing dumps to assess potential environmental impacts. Measurements were conducted in inhabited living spaces with resident consent, ensuring safe access and coverage across different areas within each settlement to reflect local geological and structural variability.

The method described in this study was intended primarily for screening purposes, allowing for the identification of areas with potential radon risks. It is important to emphasize that radon dose assessment requires long-term measurements, which provide more accurate and reliable data over time. The screening procedure used here served as an initial step in detecting areas that may warrant further investigation using long-term radon monitoring techniques to assess actual exposure levels and associated health risks.

### 2.4. Statistical Analysis

All data analysis was performed using OriginLab 2021 software to check the data distribution.

## 3. Results

### 3.1. Gamma Radiation

**Figure 2:**
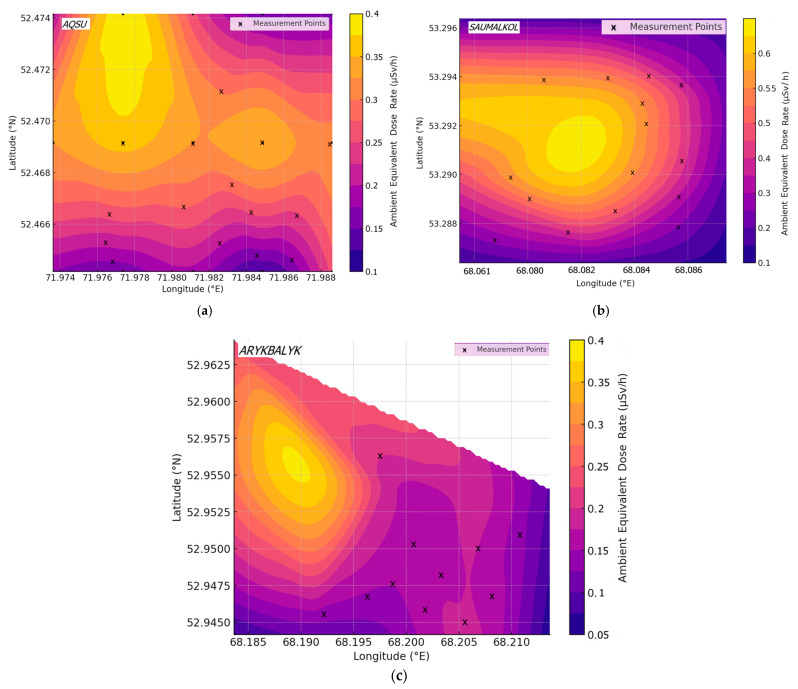
Gamma exposure dose rates in the territories of the Aqsu (**a**), Saumalkol (**b**), and Arykbalyk (**c**) settlements.

H* (10) is a critical parameter for assessing radiation exposure in different environments. In this study, measurements were taken in three settlements—Aqsu, Saumalkol, and Arykbalyk—to evaluate potential radiation risks. The measured values were compared to the intervention level of 0.12–0.16 µSv/h [25], which serves as a reference for determining whether radiation mitigation measures are necessary. The recorded H* (10) in the settlements showed variability across locations, with some measurements exceeding the intervention threshold (Figure 2).

As can be seen from Figure 2, the mean H* (10) in Aqsu (0.16 ± 0.004 µSv/h) was slightly above the intervention level (0.12 ± 0.003–0.15 ± 0.004 µSv/h), indicating the possibility of elevated radiation exposure in some areas. The maximum recorded value (0.4 ± 0.018 µSv/h) exceeded the threshold, suggesting localized hotspots. The Saumalkol location exhibited the highest variability, with H* (10) ranging from 0.10 ± 0.002 µSv/h to 0.56 ± 0.019 µSv/h. The mean H* (10) (0.27 ± 0.006 µSv/h) was significantly above the intervention level, indicating potential radiation concerns. The presence of values as high as 0.56 ± 0.019 µSv/h suggests proximity to abandoned mining areas, which could be a contributing factor. The mean H* (10) in Arykbalyk (0.15 ± 0.003 µSv/h) fell within the intervention level, suggesting that the area was relatively stable. However, individual measurements (0.13 ± 0.003–0.25 ± 0.006 µSv/h) suggest fluctuations that could be influenced by geological factors.

The Akmola region, where Aqsu is located, has a history of uranium mining, which may contribute to elevated gamma dose rates in some areas. The higher dose rates in Saumalkol could be linked to its proximity to abandoned mines, which may still release residual radioactive materials.

#### Indoor Radon Estimation

**Figure 3:**
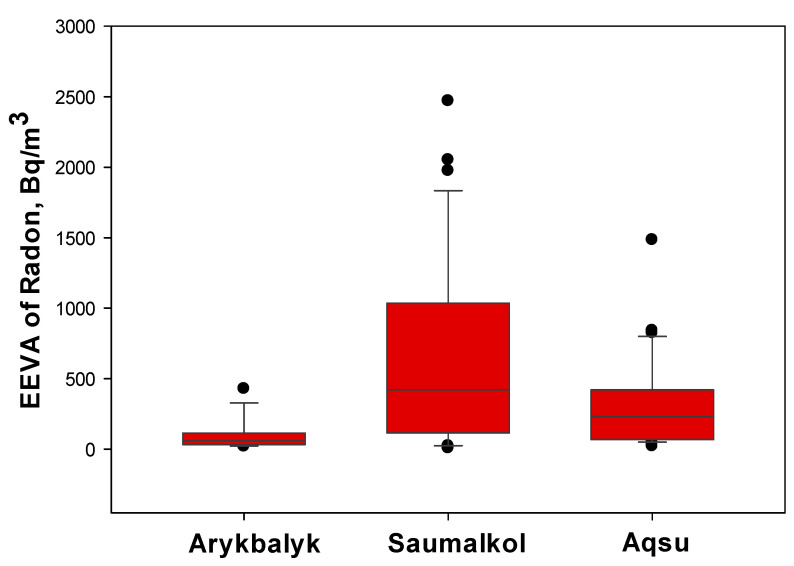
Results of the EEVA of radon concentration measurements in inhabited premises of the settlements (Bq/m^3^). The upper and lower edges of the boxes represent the 25th to 75th percentile range of radon concentration. The whiskers indicate the 90% confidence interval. The horizontal lines within the boxes correspond to the median values, while the dots represent outliers.

Radon is ubiquitously present in the air at varying concentrations and is the primary contributor to the annual effective dose. Figure 3 illustrates a comparison of the distribution of EEVA measurements for radon in the premises of the different settlements.

Figure 3 shows that the EEVA of radon levels measured in all premises in Aqsu ranged from 23 to 1485 Bq/m^3^, with a mean value of 303 ± 90 Bq/m^3^; the median was 230 Bq/m^3^, with a standard deviation of 315 Bq/m^3^. The maximum value (1485 Bq/m^3^) exceeded the permissive level according to the ICRP 37 level of 300 Bq/m^3^ [22]. This extra-high value was detected in the living room of premises that were located in the northeast part of the settlement near the sump of the former gold-mining site. In Saumalkol and Arykbalyk, the measurement results varied widely from negligible levels up to levels exceeding 300 Bq/m^3^; the mean EEVAs were 658 ± 197 Bq/m^3^ and 99 ± 30 Bq/m^3^, respectively. Median values were lower than the mean (422 and 59 Bq/m^3^, respectively), because of the outliers corresponding to the extra-high radon content in some buildings (up to 2470 Bq/m^3^ and 430 Bq/m^3^ in Saumalkol and Arykbalyk, respectively). The presence of outliers in the datasets highlights the localized nature of radon accumulation, which could be influenced by factors such as soil permeability, underground radon sources, and variations in building ventilation. The results also indicate that while radon exposure differed among settlements, there was no clear correlation with construction materials, suggesting that soil characteristics play a dominant role in determining indoor radon concentrations.

## 4. Discussion

The results of this study highlight significant variations in gamma radiation exposure and radon concentrations across the settlements of Arykbalyk, Saumalkol, and Aqsu, which are located in different uranium mining and former gold mining territories. The findings suggest that while Arykbalyk remains within a relatively lower risk category, Saumalkol and Aqsu demonstrate alarmingly high levels of radiation exposure, with potential long-term health consequences. The elevated radon concentrations and annual effective doses in these settlements significantly exceed international safety limits, necessitating urgent mitigation efforts. Gamma radiation measurements indicate that while Arykbalyk had relatively stable radiation levels within intervention thresholds, Saumalkol exhibited the highest variability, with values of up to 0.56 ± 0.19 µSv/h. Similarly, Aqsu presented localized hotspots with radiation values peaking at 0.4 ± 0.13 µSv/h. The proximity of these settlements to abandoned mining areas and tailing dumps may have contributed to the elevated exposure. Similar trends have been observed in previous studies conducted in uranium-rich regions, such as Tanzania [31] and Brazil [32], where mining activities have led to persistent environmental radiation risks.

Our findings align with research conducted by Stegnar et al. [33], which reported gamma radiation exposure levels (0.14–0.95 µSv/h) in mining regions of Kazakhstan surpassing the international safety threshold. The variability in gamma radiation in our study underscores the importance of site-specific monitoring and localized remediation efforts.

Table 2 show a comparison of indoor radon levels in uranium-mining-affected regions.

As can be seen from Table 2 the indoor radon concentrations in Aqsu and Saumalkol present severe risks, with measured values of up to 9779 Bq/m^3^ and 2471 Bq/m^3^, respectively. These figures significantly exceed the ICRP recommended limit and are consistent with findings from radon studies in high-exposure environments, such as those reported in North America [36] and in residential settings in countries of Central Asia [37], where concentrations surpassed 1000 Bq/m^3^ in mining-adjacent areas.

While this study provides valuable baseline data on environmental gamma radiation and indoor radon levels in uranium-affected settlements, it is important to recognize the limitations inherent in short-term measurements. Dose rate assessments conducted over a 40 s interval at each location offer an efficient snapshot of prevailing radiation conditions; however, they may not fully capture temporal fluctuations caused by changes in weather, soil moisture, ventilation practices, or seasonal geological activity. Similarly, radon concentrations in indoor environments can vary significantly over time, influenced by temperature gradients, occupancy patterns, and ventilation rates. As a result, single-time measurements may underrepresent or overestimate average annual exposure levels.

To improve the robustness of exposure assessments and public health risk estimates, future studies should incorporate long-term monitoring protocols. This includes the use of time-integrated dosimeters and continuous radon monitoring over multiple seasons to capture daily and seasonal variability. Long-term datasets would enable more precise estimates of annual effective dose, support the development of time-weighted exposure models, and inform more accurate calculations of excess lifetime cancer risk. Incorporating temporal variability is particularly important for regions like Northern Kazakhstan, where climate extremes and infrastructure conditions can cause wide fluctuations in indoor air quality and water usage, both of which affect radiation exposure pathways. Therefore, while our current measurements serve as a critical initial assessment, we emphasize the necessity of extended monitoring campaigns for more comprehensive radiological evaluations and health risk mitigation strategies.

## 5. Conclusions

The results of this study highlight significant radiation hazards in the studied settlements, particularly due to gamma radiation and radon exposure. The measured gamma dose rates exceeded intervention levels in multiple locations, with Saumalkol exhibiting the highest variability at 0.56 ± 0.019 µSv/h. The presence of abandoned mining sites appears to be a contributing factor to these elevated levels.

The EEVA of radon concentration measurements revealed substantial contamination, particularly in Aqsu and Saumalkol where recorded levels were 4974 Bq/m^3^ and 2470 Bq/m^3^, respectively, and significantly surpassed international safety thresholds.

These findings suggest that immediate intervention is necessary to achieve radon levels within acceptable limits, including environmental remediation efforts and public health measures to mitigate radiation exposure and associated health risks in the affected settlements.

This study presents a screening method for identifying areas with potential radon exposure risks. It is important to note that the approach used here was for screening purposes only, and that radon dose assessment requires long-term measurements to accurately evaluate exposure levels and associated health risks. Therefore, while the results provide useful insights for identifying high-risk areas, comprehensive radon dose assessment can only be achieved through extended monitoring.

## Figures and Tables

**Table 1:** Numbers and types of readings taken in various settlements in the southern region of Kazakhstan.

N	Settlement	Population	Number of Investigated Locations		
			Gamma Fields, μSv/h		Radon in the Indoor Air (EEVA, Bq/m^3^)
			Outdoor	Indoor	
1	Aqsu	4027	15	25	30
2	Arykbalyk	2850	11	11	16
3	Saumalkol	10,100	12	28	35

**Table 2:** Comparison of indoor radon levels in uranium-mining-affected regions.

Settlements	Radon Concentration, Bq/m^3^	Notes	References
Aqsu	940	High radon in homes near tailings; urgent need for mitigation	Present study
Arykbalyk	99	Moderate radon; near historical uranium site	Present study
Saumalkol	655	Close to dormant uranium mine; elevated risk	Present study
Southern Kazakhstan (Kurday)	230	Former ISL mining site; elevated radon in poorly ventilated houses	[33]
Tajikistan	15–330	Residential zones impacted by legacy uranium mining	[33]
Uzbekistan	30–600	Legacy mining impacts; many homes still lack mitigation	[33]
Kosovska, Mitrovica	90–3020	Legacy mining impacts; many homes still lack mitigation	[34]
Erongo, Namibia	400	Arid climate + fractured rock contributes to high indoor radon	[35]

## Data Availability

The data presented in this study are available upon request from the first author, Yerlan Kashkinbayev, and the correspondence author, Danara Ibrayeva.
